# Changes in the geographical and temporal patterns of cancer incidence among black gold miners working in South Africa, 1964–1996

**DOI:** 10.1038/sj.bjc.6600841

**Published:** 2003-04-29

**Authors:** N D McGlashan, J S Harington, E Chelkowska

**Affiliations:** 1 School of Geography and Environmental Studies, University of Tasmania, Hobart, Australia; 2 School of Animal, Plant and Environmental Sciences, University of the Witwatersrand, Johannesburg, South Africa; 3 School of Mathematics and Physics, University of Tasmania, Australia

**Keywords:** black gold miners, south africa

## Abstract

We describe here the results of the final 8 years of geographical and temporal data of a 33-year study of the cancer experience of 12.8 million man-years of black miners working on the gold fields of South Africa over the period 1964–96. These workers were recruited from 15 territories, the major areas during the most recent period being Lesotho (26.8%), Transkei (21.5%) and Mozambique (15%). The earliest analyses, 1964–71 and 1972–79, showed hepatocellular and oesophageal cancers to be the most frequent cancers. The final analysis, for 1989–96, however, shows marked temporal changes in the relative position of four cancers or grouped malignancies: respiratory cancer up by 236%, hepatocellular carcinoma down to 32%, oesophageal holding steady, and lymphatic system cancers up by 420%, almost certainly because of association with HIV/AIDS infection. Significant geographical variations occurring between the home areas of the miners are important, as mining operations have little to do with the cancers that develop. The causes are essentially socio-environmental rather than occupational, and this means that the rates of the major cancers in the miners are surrogate measures of the same cancers in the home areas.

This account has the two-fold object of presenting the spatial components of the final period of a 33-year study of cancers in black gold miners and of comparing these patterns with those from similar, but not identical, analyses made during previous periods. During the earliest two periods, 1964–71 and 1972–79, we hand-recorded data directly from the hospitals of the Chamber of Mines (CoM), whereas the third period, 1980–89, covered the change-over to computer record-keeping and we used the Returns to the CoM from both major and local mine hospitals. This latest analysis is from hospital-based computer records and includes cancer data from 1989 to 1996. The studies together cover 12.8 million man-years of labour, during which time some 5600 cancer cases have been diagnosed.

## MATERIALS AND METHODS

### Population-at-risk

As a result of South African government policies, the total labour force of the gold mining industry has, during the study period 1964–96, increasingly concentrated on employing men from within its national borders, and including the three ex-High Commission territories (Table 1

**Table 1:** Man-years of labour by territory as percent of all miner man-years

**Place**	**1964–68**	**1964–71**	**1972–79**	**1980–89**	**1989–96**
*South Africa*					
Cape	28.0	26.1	26.5	34.2	23.9
Transkei	NA	18.8	NA	28.2	21.5
Ciskei	NA	7.3	NA	3.1	1.2
Other Cape	NA	NA	NA	2.8	1.2
Transvaal	3.9	3.4	4.1	5.4	7.1
Free State	1.9	1.9	3.9	6.0	4.3
Kwazulu-Natal	2.6	2.3	2.8	6.3	6.6
Bophuthatswana	NA	NA	NA	3.1	7.9
					
*Ex-High Commission*					
Botswana	5.4	5.2	5.2	4.0	3.6
Swazi and Kangwane	1.4	1.5	2.0	3.3	4.8
Lesotho	15.5	16.1	22.3	24.1	26.8
					
*Foreign*					
Mozambique	23.9	24.3	17.7	10.2	15.0
Malawi and Northern territories	17.4	19.2	15.5	3.3	—
					
Total man-years	1 813 325	2 926 461	2 910 506	4 405 949	2 561 720
Average man-years p.a.	362 665	365 808	363 813	440 595	320 215

**Figure 1:**
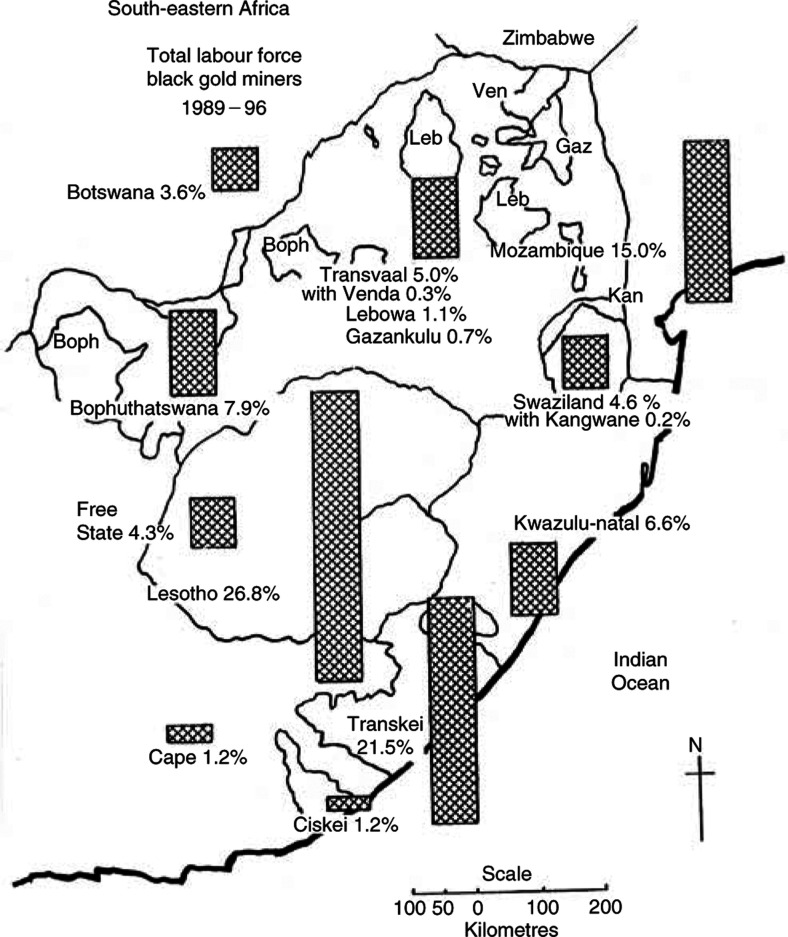
Total labour force: black gold miners by territory of origin, 1989–96.

NA=data not available.

). Mozambique has been the only exception to this policy but nonetheless has reduced numbers. The main losers were Malawi and other northern territories. The average annual total labour force has ranged from around 362 000 in 1964–71 (Harington *et al*, 1975) to 440 000 in 1980–89 (McGlashan and Harington, 2000) and 320 215 in 1989–96 (Table 1). Proportions recruited from different territories in this last period are shown in Figure 1

.

The ages of all black employees were on record at The Employment Bureau of Africa (TEBA) and varied somewhat between territories, with Mozambique providing more older men over 35 and over 45 years (Table 2

**Table 2:** Miners' age groups by percent at age, 1989–96

	**All miners**	**LES**	**TRK**	**MOZ**
**% of all**	**100**	**26.8**	**21.5**	**15.0**
15–24	8.9	6.2	5.7	5.9
25–34	42.8	36.5	47.3	24.3
35–44	31.8	35.7	31.8	42.3
45–54	13.4	17.5	12.3	21.6
55–64	3.1	4.0	2.8	5.6
65+	0.1	0.1	0.1	0.3

LES, Lesotho; TRK, Transkei; MOZ, Mozambique.

).

The names of former provinces and homelands have been used for comparability since they were in use for the greater part of this 33-year study, from 1964 to 1994.

### Cancer patients

The CoM's diagnostic policy throughout has been based upon histological confirmation, although the actual means of diagnosis is not specified in the case records. Patients diagnosed with potentially fatal malignancies may be repatriated to their homes at their own option. In the case of oesophageal cancer, clinical and radiological diagnosis would suffice for repatriation, although histology would usually have been available. Repatriated patients are recorded in both the major and local hospitals of the gold mines.

### Case finding

During the 8 years from 1 January 1989 to 31 December 1996, all cases of possible cancer were collected from the five major hospitals to which the gold mines' local hospitals and mine medical officers refer all cases of cancer. Scrupulous examination of records, even back to individual bedletters of patients, was carried out by one of us (JSH) and no available usable information, such as language or ethnicity, which could have supplemented data on region of origin, has been missed. All records were kept strictly confidential according to Ethics Clearance requirements.

The collected data included 1745 cases of primary invasive cancer. *In situ*, indefinite or doubtful cases were excluded and each multiple primary cancer was recorded as an additional case. Table 3

**Table 3:** Number and percentage of sites of cancer, 1989–96: rank and crude incidence (CIR) of each cancer per 100 000 man-years of employment

**ICD-9**	**Code**	**Site of cancer**	**Cases**	**%**	**Rank**	**CIRs**	**Recorded in 1964–79**
162	RES	Respiratory system	241	13.8	1	9.41	✓
155	HCC	Hepatocellular carcinoma	223	12.8	2	8.71	✓
150	OES	Oesophagus	198	11.3	3	7.73	✓
							
140–149	BUC	Buccal cavity	94	5.4	4	3.67	✓
202.8, 202.9	NHL	Non-Hodgkin's lymphoma	70	4.0	5	2.73	
161	LAX	Larynx	66	8.8	6	2.58	
							
153–154	COL	Colorectal	58	3.3	7	2.26	✓
204–208	LEU	Leukaemia	57	3.3	8	2.23	✓
201	HDL	Hodgkin's lymphoma	55	3.1	9	2.15	
203	MYE	Myeloma	52	3.0	10	2.03	
171	CON	Connective tissue	50	2.9	11	1.95	
151	STO	Stomach	49	2.8	12	1.91	✓
185	PRS	Prostate	49	2.8	12		
							
173	SKI	Skin	40	2.3	14	1.56	
157	PAN	Pancreas	33	1.9	15	1.29	✓
191–192	CNS	Brain and central nervous system	30	1.7	16	1.17	
171.9	KPS	Kaposi's sarcoma	28	1.6	17	1.09	
193–194	END	Thyroid, Other endocrine glands	27	1.5	18	1.05	
170	BON	Bone	23	1.3	19	0.90	
172	MEL	Melanoma	21	1.2	20	0.82	
188	BLD	Bladder	21	1.2	20		✓
189	KID	Kidney	16	0.9	22	0.62	
186	TES	Testis	15	0.9	23	0.59	
163	PLE	Pleura	14	0.8	24	0.55	
187	PEN	Penis	10	0.6	25	0.39	
152	INT	Intestine	9	0.5	26	0.35	
190	EYE	Eye	7	0.4	27	0.27	
200	LSA	Lympho- and reticulosarcoma	7	2.6	27		
175	BRS	Male breast	6	0.3	29	0.23	
195, 199, 238	USP	Ill defined, unspecified	128	7.4	—	5.04	
196–198	PSU	Metastatic but with primary site unknown	46	2.6	—	1.80	
							
a	LYS	Lymphatic system^a^	132	7.6	—	5.15	
b	HIV	HIV/AIDS-related^b^	235	13.5	—	9.17	
c	NKN	Inadequate detail/c	175	10.0	—	6.83	
							
N/N	ALL	All cancers	1745	99.9	—	68.19	

a ICD-9; 200, 201, 202.8, 202.9 are grouped together as cancers of the lymphatic system.

b Subtotals 171.9, 191–192, 201, 202.8, 202.9 and 203 as HIV/AIDS-related (see Table 9).

c Subtotals 195, 196–199 and 238 as inadequate detail/not known.

gives the number of cases, percentage frequency, rank and crude incidence rates for individual sites and types of cancer. Of the records for the 1745 total cases, 521 were in some measure incomplete. Those with no address were treated as ‘territory not known’ (TNK), and the numbers have been quoted in all the tabulations of incidence. There is no reason to suppose that they are drawn disproportionately from any particular territory. Those with age at diagnosis unknown were distributed *pro rata* to the known age distribution of cases with that same diagnosis. Table 4

**Table 4:** Age groups of miners at diagnosis in 1964–71 and 1989–96 for respiratory (RES), hepatocellular (HCC), oesophageal (OES) and bladder cancers (BLD)

	**RES**		**HCC**		**OES**		**BLD**	
Age (years)	**1964–71^a^**	**1989–96**	**1964–71^a^**	**1989–96**	**1964–71^a^**	**1989–96**	**1964–71^a^**	**1989–96**
15–24	1.4	0.0	17.9	0.9	0.6	0.0	7.7	0.0
25–34	9.6	2.5	34.8	14.3	8.0	5.1	20.0	4.8
35–44	27.4	18.3	21.3	18.8	26.5	17.2	29.2	19.0
45–54	31.5	39.8	17.3	24.7	42.6	32.8	30.7	9.5
55+	19.2	17.8	4.1	14.3	18.6	20.7	10.8	19.0
NKN	10.9	21.6	4.6	26.9	3.7	24.2	1.6	47.6
								
Median age	47.0	49.6	33.6	46.0	48.0	49.8	42.4	47.5

a After Harington *et al* (1975); NKN=Age not known.

shows that the median age at which the four major cancers originally studied were diagnosed has risen, especially for hepatocellular carcinomas.

### Analyses of geographical and temporal patterns of cancer

Using the overall age-specific rates for all miners (in 5-year age-groups from 15–19 to 64+) as the standard population, expected numbers have been derived for the individual territories and districts and used to calculate age-standardised incidence ratios (ASIRs) (Table 5

**Table 5:** Geographical and temporal variations of major sites of cancer and of all malignancies among black gold miners by home territory, 1964–79 and 1989–96

			**Respiratory**					**Hepatocellular**					**Oesophagus**					**Buccal cavity**				
Place	**Population**		**CIR**	**CIR**	**Obs**	**Exp from**	**ASIR**	**CIR**	**CIR**	**Obs**	**Exp from**	**ASIR**	**CIR**	**CIR**	**Obs**	**Exp from**	**ASIR**	**CIR**	**CIR**	**Obs**	**Exp from**	**ASIR**
	**1989–96**	**%**	**1964–79**	**1989–96**	**1989–96**	**1964–79**	**1989–96**	**1964–79**	**1989–96**	**1989–96**	**1964–79**	**1989–96**	**1964–79**	**1989–96**	**1989–96**	**1964–79**	**1989–96**	**1964–79**	**1989–96**	**1989–96**	**1964–79**	**1989–96**
*South Africa*																						
Cape	613 167	23.9	4.8	13.0	80	29.2++	148++	11.5	8.8	54	70.5−	105	15.2	16.5	101	93.2	231++	1.1	5.2	32	6.7++	151
TrK	550 256	21.5		11.6	64		130		8.4	46		99	14.6	14.9	82		206++		4.7	26		134
CiK	31 075	1.2		16.1	5		200		9.7	3		122		9.7	3		149		3.2	1		102
Oth	31 836	1.2		34.6	11		485++		15.7	5		212		50.3	16		860++		15.7	5		549
TVL	183 798	7.1	7.7	6.0	11	14.2	81	9.3	3.8	7	17.1−	49−	7.7	7.6	14	14.2	120	1.0	2.2	4	1.9	71
FS	111 381	4.3	4.3	15.3	17	4.8++	244++	2.5	6.3	7	2.8	94	9.2	9.9	11	10.2	189	0.0	8.1	9	0.0++	319+
KZN	167 911	6.6	10.2	4.8	8	21.4−−	141	27.2	6.6	11	45.7−−	135	10.2	7.1	12	17.1	239+	2.6	4.2	7	4.3	278
BOP	202 142	7.9		4.0	8		93		1.0	2		18−−		2.5	5		67		1.0	2		53
																						
*Ex-High Commission*																						
BOT	91 079	3.6	1.1	2.2	2	1.0	22−−	2.7	1.1	1	2.5	12−−	3.0	3.3	3	2.7	41	0.8	0.0	0	0.7	0
SWZ & KAN	121 959	4.8	2.4	2.5	3	2.9	46	4.8	6.6	8	5.9	105	1.2	0.0	0	1.5	0−−	0.0	0.0	0	0.0	0
LES	686 770	26.8	1.9	9.3	64	13.3++	79−	5.2	2.8	19	35.7−−	28−−	2.3	2.5	17	15.8	26−−	0.5	2.2	15	3.7++	49−−
																						
*Foreign*																						
MOZ	383 513	15.0	1.9	4.7	18	2.2++	32−−	57.0	17.0	65	218.6−−	142+	1.3	1.3	5	5.0	11−−	0.4	2.1	8	1.6	38−−
TNK					30					49					30					17		
																						
Total	2 561 720	100	3.1	9.4	241	78.6++	100	18.8	8.7	223	473.3−−	100	6.3	7.7	198	160.1++	100	0.7	3.7	94	16.9++	100
	**Colorectal**					**Leukaemia**					**Larynx**			**Stomach**				**All malignancies**				
Place	**CIR**	**CIR**	**Obs**	**Exp from**	**ASIR**	**CIR**	**CIR**	**Obs**	**Exp from**	**ASIR**	**CIR**	**Obs**		**CIR**	**CIR**	**Obs**	**Exp from**	**CIR**	**CIR**	**Obs**	**Exp from**	**ASIR**
	**1964–79**	**1989–96**	**1989–96**	**1964–79**	**1989–96**	**1964–79**	**1989–96**	**1989–96**	**1964–79**	**1989–96**	**1989–96**	**1989–96**	**ASIR**	**1964–79**	**1989–96**	**1989–96**	**1964–79**	**1964–79**	**1989–96**	**1989–96**	**1964–79**	**1989–96**
*South Africa*																						
Cape	1.1	1.5	9	6.7	67	0.9	2.9	18	5.3++	134	4.1	25	139+	1.4	2.0	12	8.6	41.4	78.0	478	253.9++	123++
TrK		1.1	6		49		2.9	16		132	3.6	20	152		1.8	10			72.3	398		110
CiK		3.2	1		155		0.0	0		0	9.7	3	452		0.0	0			61.1	19		102
Oth		6.3	2		317		6.3	2		313	6.3	2	330		6.3	2			191.6	61		341++
TVL	1.1	2.7	5	1.8	128	0.5	0.6	1	0.9	26	2.7	5	132	1.0	1.1	2	1.9−	36.0	54.4	100	66.2++	92
FS	0.0	3.6	4	0.0	200	0.0	3.6	4	0.0	190	1.8	2	107	0.6	1.8	2	0.7	19.7	93.4	104	21.9++	184++
KZN	2.6	0.6	1	2.8	44	0.9	0.6	1	1.4	37	1.2	2	131	4.2	1.8	3	7.1	73.0	48.2	81	122.6−−	138+
BOP		1.0	2		63		2.0	4		119	0.0	0	0		0.0	0			18.3	37		45−−
																						
*Ex-High Commission*																						
BOT	0.8	2.2	2	0.7	98	1.1	1.1	1	1.0	47	2.2	2	83	1.1	0.0	0	1.1	14.8	28.5	26	13.5+	40−−
SWZ & KAN	0.0	0.0	0	0.0	0	0.0	3.3	4	0.0	180	1.6	2	116	0.0	1.7	2	0.0	16.7	31.2	38	20.4++	67−−
LES	1.1	1.6	11	8.1	63	1.2	0.9	6	8.1	35−−	2.0	14	63	1.0	2.2	15	6.9+	17.2	50.0	343	118.1++	62−−
																						
*Foreign*																						
MOZ	0.6	2.9	11	2.4+	95	0.8	2.6	10	3.2+	91	1.8	7	44−−	0.5	0.5	2	1.9	73.3	54.0	207	281.1−−	56−−
TNK			13					8				7				11				331		
																						
Total	0.9	2.3	58	22.3++	100	1.0	2.3	57	26.6+	100	2.6	66	100	1.0	1.9	49	25.4++	38.2	68.2	1745	978.6++	100

Poisson significance increase : *P*<0.05+; *P*<0.01++. Decrease: *P*<0.05−; *P*<0.01−−.

TNK=home territory not known.

).

Since only crude incidence rates (CIRs) are available from previous periods, time changes of specific cancers (by place) have been calculated using the CIRs of 1964–79 as the standard, excluding 1969–71 for which data are no longer available (McGlashan *et al*, 1982), and applying these to the 1989–96 populations to produce expected numbers. The intervening period 1980–89 is ignored because its database was differently derived and covered merely three sites of cancer. Table 5 shows, by individual territories, for eight of the most common cancers and for all malignancies, the CIRs for 1964–79 and 1989–96 together with the observed values for 1989–96, expected values from 1964 to 1979, and ASIRs for 1989–96. (Only geographical comparisons are available for cancer of the larynx and only temporal comparisons are given for cancer of the stomach, which had fewest numbers.) All CIRs throughout the paper are expressed per 100 000 population at risk. Significant geographical and temporal deviations are marked in the table from Poisson's comparison of observed and expected values. The cases with territory not known have not been proportionally distributed between the local areas, because this would have artificially inflated the levels of significance without increased accuracy. The local rates presented are therefore minimal rates. Tabulation of the numbers that have not been included for each type of cancer means that the degree of possible local underreporting can be assessed (Table 5).

Geographical comparisons for 1989–96 are presented in Table 6

**Table 6:** Regions of Mozambique: hepatocellular carcinoma CIRs, ASIRs and significance, 1989–96

**District**	**Popln.^a^**	**CIR**	**Obs.**	**Exp.^b^**	**ASIR^c^**	**Signif.^d^**
Bilene	27 350	3.7	1	3.3	30.6	
Vilanculos	23 766	4.2	1	2.8	35.3	
Xai-Xai	63 523	4.7	3	7.6	39.6	−−
Chibuto	34 359	8.7	3	4.1	73.2	
Guijá	29 550	10.2	3	3.5	85.1	
Maputo	64 547	9.3	6	9.8	91.9	
Matola	17 533	17.1	3			
Panda	30 140	19.9	6	3.6	171.2	
Homoine						
Inharrime						
Manhica	22 547	22.2	5	2.7	185.8	
Magude						
Inhambane	36 017	33.3	12	4.3	279.2	++
Murrumbene						
Massinga						
Zavala	34 181	41.0	14	4.1	343.2	++
Muchopes						
*District not known*			8			

**Mozambique**	**383 513**	**16.9**	**65**	**45.8^e^**	**141.9^f^**	**++**

a Population in man-years of labour employment.

b Expected number calculated at Mozambique truncated age-standardised incidence ratio (ASIR) 141.9.

c Local ASIR for the district.

d Observed greater than expected value at ++*P*<0.01 by Poisson distribution. Observed less than expected value at −−*P*<0.05.

e Expected total calculated on all miners' rate.

f ASIR calculated on all miners as 100.

and Table 7

**Table 7:** Districts and regions of Transkei: oesophageal cancer CIRs, ASIRs and significance, 1989–96

**Region**		**District**	**Popln.^b^**	**CIR**	**Obs.**	**Exp.^c^**	**ASIR^d^**	**Signif.^e^**
OFS	28	Herschel^a^	16 876	35.6	6	1.2	491.8	
								
	10	Matatiele		8.6	4			
	11	Mt Ayliff		0	0			
	12	Mt Fletcher		11.5	2			
	13	Mt Frere		22.2	4			
	18	Qumbu		7.6	1			
	21	Tsolo		5.0	1			
	24	Umzimkulu (not included)						
								
East Griqualand			121 749	9.9	12	8.8	136.4	
								
	1	Bizana		12.9	5			
	5	Flagstaff		4.3	1			
	8	Libode		4.1	1			
	9	Lusikisiki		9.9	2			
	15	Ngqeleni		15.1	4			
	17	Port St Johns		27.9	3			
	20	Tabankulu		6.9	2			
								
Pondoland			116 557	15.4	18	8.4	213.6	−−
								
	3	Elliotdale		40.4	4			
	4	Engcobo		3.8	1			
	14	Mqanduli		0	0			
	19	Cofimvaba		20.0	3			
	23	Umtata		11.4	2			
	26	Xalanga		31.8	4			
	27	Lady Frere^a^		18.6	3			
								
Tembuland			192 848	8.8	17	13.9	122.0	
								
	2	Golwa (Butterworth)		25.1	3			
	6	Idutywa		18.3	4			
	7	Kentani		34.4	6			
	16	Nqamakwe		26.0	5			
	22	Tsomo		8.0	1			
	25	Willowvale		26.0	5			
								
Transkei Unit			102 226	23.5	24	7.4	324.8	++
		*District not known*			5			
**Transkei**			**550 256**	**14.9**	**82**	**39.8^f^**	**206.2^d^**	++^*^

District numbering on the map (Figure 3) follows the earlier mapping (Rose and McGlashan, 1975).

a Herschel and Lady Frere districts were not included as parts of Transkei in 1964–79.

b Population in man-years of labour employment.

c Expected on the basis of Transkei truncated age-standardised incidence ratios (ASIR) 206.2.

d Local ASIR for the region.

e Significance (by Poisson distribution) high at ^++^*P*<0.01, high at ^−−^*P*<0.05.

f Expected total calculated on all miners' rate.

for regions and districts within Mozambique (hepatocellular carcinoma) and Transkei (oesophageal cancer). Table 8

**Table 8:** Geographical and temporal variations of HIV/AIDS-related cancers

		**NHL (200+202)**		**HDK (201)**		**MYE (203)**		**CNS**		**KPS**		**TOTAL HIV**		**HIV-AIDS**		**200–203**	
Place	**Total labour force 1989–96**	**Obs**	**Exp**	**Obs**	**Exp**	**Obs**	**Exp**	**Obs**	**Exp**	**Obs**	**Exp**	**Obs**	**Exp**	**ASIR**	**CIR**	**Obs**	**Exp from 1964–71**
*South Africa*																	
Cape	613 167	12	16.2	11	13.2	10	12.5	5	7.2	2	6.5	40	54.6	73−	6.5	33	6.2++
Transkei	550 256	11	14.6	8	12.0	9	10.4	5	6.4	2	5.9	35	49.4	71−	6.4	28	
Ciskei	31 075	0	0.8	0	0.6	0	0.5	0	0.4	0	0.3	0	2.6	0	0	0	
Other Cape	31 836	1	0.8	3	0.7	1	0.5	0	0.3	0	0.3	5	2.6	193	15.7	5	
Transvaal	183 798	7	4.9	2	3.9	4	3.6	1	2.0	0	1.7	14	15.8	89	7.6	13	3.8++
Free state	111 381	4	2.6	2	2.1	4	1.6	6	1.1+	0	0.9	16	8.3	192+	14.4	10	1.4++
Kwazulu-Natal	167 911	5	3.4	6	2.7	4	1.4	3	1.4	0	1.0	18	9.8	183	10.7	15	4.3++
Bophuthatswana	202 142	1	4.2	0	3.5	1	2.2	0	1.8	0	1.3	2	12.9	16−−	0.9	2	

*Ex-High Commission*																	
Botswana	91 079	1	2.6	0	2.0	0	1.9	1	1.1	3	1.1	5	8.8	57	5.5	1	0.3
Swazi and Kangwane	121 959	3	2.7	1	2.3	1	1.5	0	1.2	0	0.0	5	7.8	64	4.1	5	2.9
Lesotho	686 770	14	20.2	16	15.6	15	16.6	9	8.8	1	8.7−	55	69.9	79	8	45	4.4++

*Foreign*																	
Mozambique	383 513	6	13.2−	9	9.6	5	12.0−	3	5.4	7	5.8	30	46.1	65−−	7.8	20	7.6+
TNK		17		8		8		2		15		50				33	

Total	2561 720	70		55		52		30		28		235			9.2	177	34.1++
CIR		2.7		2.1		2.0		1.2		1.1		9.2				6.9	

Significance levels as Table 5.

shows the geographical distribution of five malignancies that have been related to HIV and AIDS (Goedert *et al*, 1998).

## RESULTS

### Spatial and temporal distribution of cancer sites

*Cancer of the respiratory system (ICD 9, 162)*

*Results*. This is now the most numerous site of cancer in the total labour force of the gold mining population with 241 cases (Table 5). Two territories, Cape and Free State, record significantly above the pooled CIR of 9.4, with ASIRs of 148 and 244, respectively. Significantly few cases were found in men recruited from Mozambique, Botswana and Lesotho. Lesotho, with a large work force, had 64 cases against 80.6 expected and an ASIR of only 79.4. Only one district within Lesotho, miners from Leribe, had significantly (*P*<0.01) fewer cases than expected from Lesotho rates.

Respiratory cancer has increased sharply and significantly in the total labour force although not among miners from Transvaal and Kwazulu-Natal, where the rates were previously relatively high, and not in Swaziland, where they have remained low. In Kwazulu-Natal, the most recent figures indicate a significant fall in incidence (*P*<0.01) (Table 5).

*Discussion*. The median age of miners with respiratory cancer (Table 4) has increased only slightly, from 47.0 in the 1960s to 49.6 in 1989–96, and the increases in CIRs that have been observed are most likely genuine and not a reflection of increasing age of the mining population. Cigarette smoking particularly, and also exposure to dust in mines, are proven causal factors (Wyndham *et al*, 1986). Greater accessibility for the miners of commercial cigarettes is likely because of their higher earning power in recent times. There are also areas where the custom of smoking Western cigarettes has followed the pre-existing smoking of native tobacco with this probably facilitating the uptake of the new habit. This could help explain the high rates among the Xhosa, who are the most numerous people in both high-incidence territories (Bradshaw *et al*, 1983). Previous evidence of ‘copy-cat’ acquisition of a Western trait in urban centres (McGlashan and Harington, 1985) is neither supported nor denied by the current findings as the miners were mainly recruited from rural areas.

Early evidence had pointed to the Zulu being especially affected by this cancer (Robertson *et al*, 1971), whereas nowadays Kwazulu-Natal rates (CIR 4.8) fall well below the average rate for all miners (9.4) and below half their own rate of 12.7 in 1964–79 (McGlashan *et al*, 1982).

*Hepatocellular carcinoma (HCC) (ICD 9, 155)*

*Results*. This is the second most common cancer among gold miners with 223 cases, but has dropped from being the most common site in 1964–68, when it represented 56.2% of all cancers among gold miners (Robertson *et al*, 1971), to only 12.8% in 1989–96 (Table 3). While only 15% of the labour force came from Mozambique in 1989–96, 29% (65) of cases were among these men (ASIR 142; *P*<0.05) (Table 5). Transkei had the next highest number of cases (46) but an ASIR of 99 and a CIR of 8.4, effectively equal to the pooled rate for all miners (CIR 8.7). A significantly low incidence of HCC was observed for Bophuthatswana, Botswana and Lesotho (*P*<0.01) and the Transvaal (*P*<0.05).

The CIRs for HCC in 1989–96 are mostly lower than those for 1964–79 and all the significant changes are in this direction (Table 5). Table 4 shows the median age for HCC as 46.0 years compared with 33.6 years at 1964–71. The average age at diagnosis was already rising sharply by 1979, and Bradshaw *et al* (1982) suggested that ‘some basic improvement in general living conditions’ must underlie this change.

**Figure 2:**
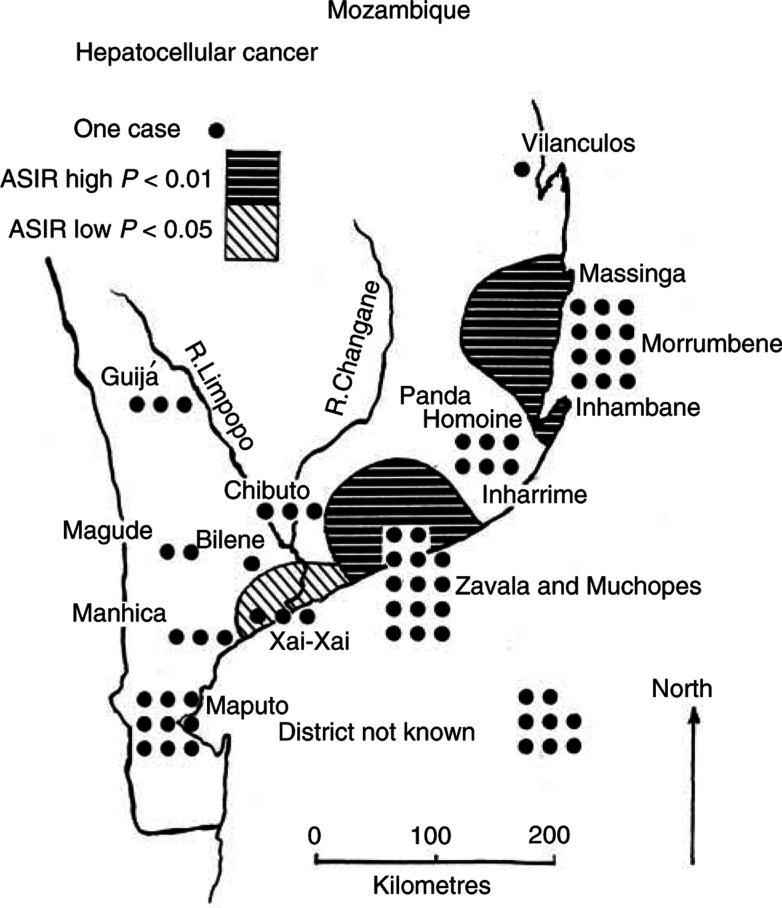
ASIR for hepatocellular carcinoma in Mozambique.

The local distribution of HCC within Mozambique in 1989–96 (Figure 2

, Table 6) shows the highest rates concentrated in the coastal belt from Muchopes to Massinga, with values in the grouped Zavala and Inhambane areas that are significantly above the level for the territory (ASIRs of 343 and 279, respectively, *P*<0.01 for both areas). Gold miners from these districts also had significantly high rates (*P*<0.01) in 1964–79 (Bradshaw *et al*, 1982). In the current study, miners from the valley of the Limpopo River represented by Bilene, Xai Xai, Chibuto and Guijá have considerably lower ASIRs (31, 40 (*P*<0.05), 73, 85, respectively). The lower numbers in the current study, because of falling rates, mean that fewer areas have rates that diverge significantly from the regional average than in previous periods. The CIRs for the Mozambique districts now range from 3.7 to 41.0 per 100 000 man-years and are, at the top end of the scale, only about a quarter of what occurred in 1964–71 (Harington *et al*, 1975). However, the enduring geographical pattern within the territory remains very much as it was in the earlier years (Bradshaw *et al*, 1982).

*Discussion*. The most serious epicentre of HCC in southern Africa is in Mozambique, where the condition has long been noted as a problem, affecting both men and women (Prates and Torres, 1965). Recent local evidence from Mozambique (Pers. commun. Ministério da Saúde, Maputo, 2001), as well as the marked decrease in HCC among expatriate gold miners since 1964–71 and the rise in the average age at which the disease develops, all suggest that a pronounced change in environmental factors has occurred during the last 30 years (Table 5). However, the enduring geographical pattern within the territory may also imply more permanent differences of climate or of food supplies and diet between the coastal areas to the east and the Limpopo Valley.

Chronic infection with hepatitis-B virus and ingestion of mycotoxins, especially aflatoxin, from groundnuts poorly stored in a hot, humid environment have been shown to be risk factors in various studies from Africa and Asia (London and McGlynn, 1996). However, it is difficult to envisage that the prevalence of either of these would have changed favourably during recent times of famine and civil war in Mozambique (Harington *et al*, 2002). Comparison could usefully be made with the neighbouring peaceful country of Swaziland, which has had, and may still have, high rates of aflatoxin intake and of HCC among its population (McGlashan, 1982) and where rates of HCC have recently risen (Table 5).

*Carcinoma of the oesophagus (ICD 9, 150)*

*Results*. For this cancer in 1989–96 (Table 5), half of all cases with known home territory (82 out of 168) were from the Transkei, giving an ASIR of 206 (*P*<0.01) relative to the CIR of 7.7 for the total labour force. Significantly high rates were also observed in regions of the Cape to the west and north-west of Transkei and Ciskei (*P*<0.01) and in Kwazulu-Natal (*P*<0.05). The exceptionally high ASIR for ‘Other Cape’ must be viewed against generally high ASIRs for all cancers for these territories suggesting that they may include a number of Cape residents whose address is not further specified. Nonetheless, it indicates at least a two-fold genuine excess. Miners with significantly low numbers were those from the more distant territories of Mozambique (ASIR 11), Lesotho (ASIR 26) and Swaziland (no cases) (*P*<0.01 for all three areas).

The rates in Transkei have been the highest of any territory since the inception of these studies of cancer among gold miners, but have remained virtually unchanged over the two time periods considered here. The increase of 22% in the pooled CIR has occurred because of a wider spread of the disease beyond Transkei itself. Recruits from the western Cape are predominantly Xhosa, like those from the Transkei, while the Zulu occupy neighbouring territories and share some customs with the Xhosa (see the ethnic map in McGlashan and Harington, 1985). Miners from Mozambique, Lesotho and Swaziland are respectively, for the most part, Shangaan, Basuto or Swazi people so that geographical differences for cancers at this site are likely to reflect customs that differ between the different groups, such as abuse of alcohol and tobacco products.

**Figure 3:**
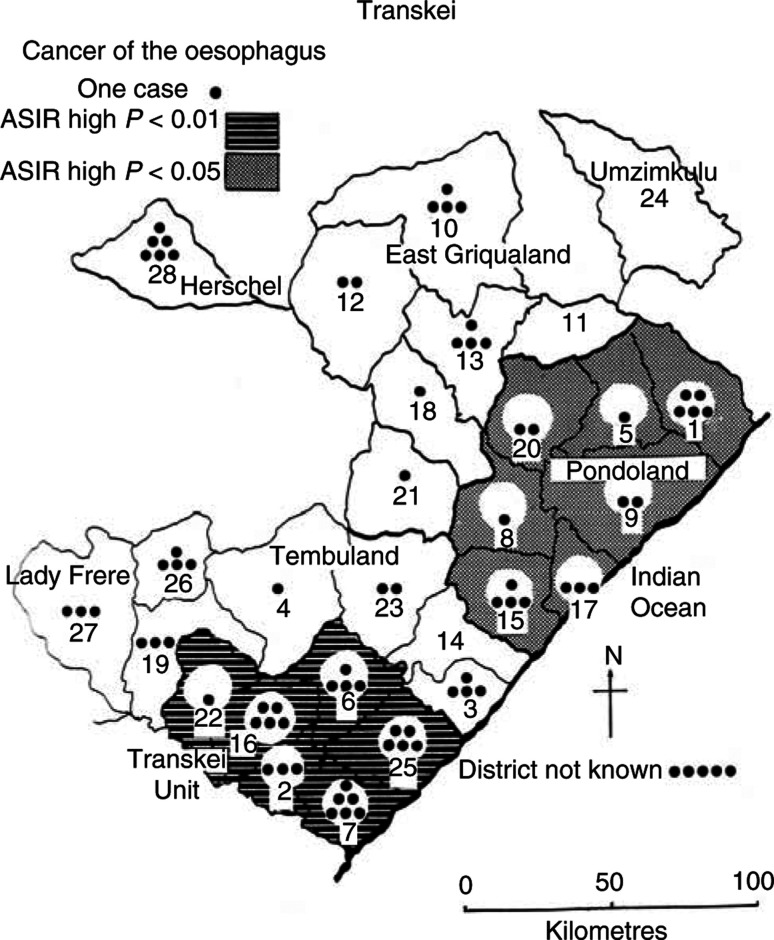
ASIR for distribution of oesophageal cancer in regions of Transkei.

Earlier analyses of rates for the periods 1964–79 among miners from the four regions within Transkei and also for individual districts (Bradshaw *et al*, 1982) showed a significant excess of cases in the southern region, Transkei Unit (*P*<0.05), and a deficit in the northern region, Pondoland (*P*<0.01), compared with the rates for the whole of Transkei. The current data also permit examination of the internal distribution of cancer cases among miners from Transkei for 1989–96 (Figure 3

, Table 7). The CIR for the Transkei Unit itself now has hardly changed, 23.5 against 23.3 earlier; in Tembuland and East Griqualand rates have decreased (though not significantly) to CIRs of 9 from 18 and 15. The decrease has been especially marked in Umtata, the capital of Transkei, and adjacent districts of Mqanduli and Engcobo. Less satisfactory, however, is the increase to a relatively high CIR of 15.4 (*P*<0.05) in Pondoland, where the condition was previously significantly low.

As with respiratory cancers, there has been only a slight rise in the median age at diagnosis for oesophageal cancer (48.0 in 1964–71 to 49.8 most recently), probably in line with the increasing age of the total labour force (Table 4).

*Discussion*. Over the years, this site of cancer has been recognised as one of the major health problems of Transkei, both from studies of the gold miners and of men and women resident in the territory (Rose and McGlashan, 1975). From its geographical distribution, it has been firmly associated with Xhosa customary usage of alcohol and homegrown and commercial tobacco, although there seems little doubt that the use of pipe tobacco in various forms is the principal determinant (Bradshaw *et al*, 1983).

What now seems significant is that oesophageal cancer is no longer solely concentrated in Transkei, where rates are virtually unchanged, but has spread more broadly outside that territory.

*Cancer of the buccal cavity (ICD 9, 140–149)*

*Results*. Significantly more cases of cancer of the buccal cavity arise in miners from the Free State (*P*<0.05) and significantly few in miners from Lesotho and Mozambique (*P*<0.01) (Table 5). Elevated ASIRs are also found in all three Cape subareas and in Kwazulu-Natal, emphasising a major contrast between miners from the Xhosa and Zulu areas compared with Basuto and Shangaan miners.

This site of cancer, which was rarely seen in the earlier period, has increased in occurrence from a pooled CIR of 0.7 in 1964–79 to 3.7 in 1989–96 (Table 5). The increase is particularly marked among miners from the Cape, Free State and Lesotho, which have all seen a rise in the number of cases that is more than four-fold (*P*<0.01 for each territory) (Table 5).

*Discussion*. Other data sources show that this cancer is also common among platinum miners recruited from Bophuthatswana and the northern parts of the Cape (McGlashan *et al*, 2002). The major established risk factors, apart from the chewing of betel nut, which is not practised among black South Africans, are tobacco, including chewing and the use of snuff (Blot *et al*, 1996), and alcohol. The recent increase may, as with lung cancer, relate to the greater use of tobacco in various forms among miners.

*Cancer of the larynx (ICD 9, 161)*

*Results*. Laryngeal cancer, with a pooled CIR of 2.6, is significantly more common among miners from the Cape (ASIR 139, *P*<0.05) than in those from other territories. Mozambique (*P*<0.01), Lesotho and Bophuthatswana recorded few cases (Table 5). In 1964–79, larynx was included with ‘other cancers’ and so no previous data are available for temporal comparisons.

*Discussion*. Cancer of the larynx has a proven aetiological association with the use of alcohol and smoking.

*Colorectal cancer (ICD 9, 153–154)*

This cancer is of low incidence generally even though there has been an increase in the pooled CIR from 0.9 in 1964–79 to 2.3 in 1989–96 (*P*<0.01). It is especially low in Transkei, with an ASIR of 49, and has risen in Mozambique (from CIR 0.6 to 2.9 (*P*<0.01) (Table 5). As with Mozambican rates of hepatocellular cancer, this change may imply a diet-related aetiology.

*Leukaemia (ICD 9, 204–208)*

*Results*. Leukaemia has a pooled CIR of 2.3 doubled from 1.04 in 1964–79. It shows little indication of geographical variation apart from a low ASIR in Lesotho (ASIR 35, *P*<0.01) (Table 5). The major rise in incidence of leukaemia, especially in the Cape and Mozambique, is contemporaneous with the major onset of HIV/AIDS in southern Africa.

*Stomach cancer (ICD 9151)*

There is little significant spatial variation of CIRs for this cancer, although the pooled CIRs have nearly doubled from 0.99 to 1.9 (*P*<0.01). Much of the increase has occurred in miners from Lesotho.

*Bladder cancer (ICD 9, 158)*

This cancer now has a pooled CIR of 0.8 (Table 3) compared with 1.5 in 1964–79 (*P*<0.01) (Bradshaw *et al*, 1982). As with HCC, it was formerly most common in Mozambique with a CIR of 4.3 in the earlier period. However, the recent CIR there is significantly below the pooled rate for all miners (*P*<0.01).

*HIV/AIDS-related cancers*

*Results*. The five cancers that are related to HIV infection (non-Hodgkin's lymphoma, Hodgkin's lymphoma, myeloma, central nervous system and Kaposi's sarcoma) (Sitas *et al*, 1997; Goedert *et al*, 1998) together total 235 cases (Table 8). This is too few for any clear indication of geographical variation in the individual sites and types of cancer. However, for the grouped sites, Free State has almost twice the expected number (ASIR 192, *P*<0.05) and Bophuthatswana less than one-fifth (*P*<0.01). The numbers are also relatively low in Mozambique (*P*<0.05) (especially for NHL and myeloma) and in Transkei (*P*<0.05). In Lesotho, only one case of Kaposi's sarcoma was recorded compared with nine expected. The pooled CIR of 9.2 places this group of cancers in rank second only to respiratory cancer. No comparable data exist for earlier periods to provide a temporal comparison with the total group of AIDs-related malignancies, but for the lymphomas and myeloma the pooled CIR has increased five-fold from 1.3 in 1964–79 (McGlashan *et al*, 1982) to 6.9 in 1989–96 (Table 8) and most territories have experienced a higher number of cases.

*Discussion*. South Africa is facing one of the most severe epidemics of HIV in the world (Department of Health, 1999), and miners have certainly not escaped infection. Gilgen *et al* (2001) report that in one major gold-mining complex 22% of men in the township and 29% of miners were infected. Thirty-seven per cent of women in the same community and 69% of ‘sex workers’ were HIV positive. Rather than reflecting the rates of the home territories, for these cancers, the miners may well incur infection while working at the mines and then be instrumental in the spread of HIV and the related cancers whenever they return home. To some extent, the spread may depend on the strength of religious views and on the taboos of morality that are practised in different territories.

*All sites of cancer*

*Results*. The figures for all malignancies in Table 5 emphasise that three territories (Cape Province, Free State and Kwazulu-Natal) have a cancer burden that is significantly above the pooled rate for all territories of 68.2. All three are peopled by the related Xhosa and Zulu groups, in contrast to the Shangaan, Sotho and Tswana elsewhere (McGlashan and Harington, 1985). Areas to the east (Mozambique) and northwest (Botswana and Bophuthatswana) have lower case numbers than would be expected, as has the largest provider of mine workers, Lesotho.

Overall, there had been a 1.8-fold increase in the CIRs for ‘all cancers’ between the two study periods. This has been contributed to largely by respiratory and, to a lesser extent numerically, by buccal cavity cancers and has occurred despite the general fall in numbers for liver cancer (Table 5). The increases appear to reflect genuine changes in incidence rates and not to be because of any major upward shift in the age of the mining population (Table 4). Only two territories, Kwazulu-Natal and Mozambique on the Indian Ocean coast, have significantly improved their cancer experience, largely because of the fall in numbers for liver cancer (Table 5).

## CONCLUSION

A major benefit of the earlier cancer surveys among gold miners was the clear demonstration that the spatial and temporal patterns of the two major cancers of the miners, hepatocellular and oesophageal, served as surrogate measures for the distribution and frequency of these same cancers in the territories of origin of the miners. This was particularly so given that the miners have largely been recruited from rural areas and that these two cancers have certain causal factors that are more common in relatively poor, traditional societies. For respiratory, buccal cavity and laryngeal cancer, the low initial rates among the miners from many territories highlight those areas where commercial cigarettes have not yet, or have only recently, penetrated.

The clear geographical indicators derived from the earlier studies permitted two successful field studies to be undertaken. One was in Transkei on oesophageal cancers in which the uses of pipe tobacco and alcohol in various ways were found to be the principal aetiological factors (Bradshaw *et al*, 1983), albeit with a strong indication that underlying nutritional deficiencies also had an important role (Van Rensburg, 1981). The second concerned the ingestion of aflatoxin as one cause of hepatocellular carcinoma in Mozambique (Van Rensburg *et al*, 1985).

This paper brings to a close a long series of studies by the same research team of cancer incidence among black gold miners from southern Africa and highlights both enduring and new geographical and temporal patterns of occurrence. It is hoped that these will lead to the instigation of new aetiological investigations in the territories or regions that we have shown to be of particular interest.
