# Changes in the geographical and temporal patterns of cancer incidence among black gold miners working in South Africa, 1964–1996

**DOI:** 10.1038/sj.bjc.6601174

**Published:** 2003-08-12

**Authors:** N D McGlashan, J S Harington, E Chelkowska

## Abstract

**Correction to**: *British Journal of Cancer* (2003) **88**, 1361–1369. doi:10.1038/sj.bjc.6600841

A couple of errors have been noted as follows:

In Table 6 (on p 1366), the significance value for Xai-Xai should be shown as a single minus and in its footnote as ‘observed less than expected value at −*P*<0.05’.

Similarly in Table 7 (on p 1367), the significance value for Pondoland should be shown as a single plus and in its footnote as ‘high at +*P*<0.05’.

The Publisher would like to apologise for any inconvenience this may have caused.

